# Bullous pemphigoid in a patient with a neuropsychological disorder and a possible novel drug trigger: A case report and review of the literature

**DOI:** 10.1002/ski2.176

**Published:** 2022-10-21

**Authors:** Sarah M. Dyson, Priya U. Patel, Laszlo Igali, George W. M. Millington

**Affiliations:** ^1^ Dermatology Department Norfolk and Norwich University Hospital Colney UK; ^2^ Norwich Medical School, UEA Norwich UK; ^3^ Cellular Pathology Department Norfolk and Norwich University Hospital Colney UK

## Abstract

A 59‐year‐old woman with schizoaffective disorder presented with an itchy, blistering generalised rash. One month prior, she had started empagliflozin, a sodium glucose transporter‐2 (SGLT‐2) inhibitor, used in type‐2‐diabetes. She was already established on paliperidone, an atypical antipsychotic, for 1 year. Serology at presentation was positive for anti‐pemphigoid antibodies. Histology demonstrated subepidermal blistering, perivascular inflammation and eosinophils. Direct immunofluorescence was characteristic of bullous pemphigoid (BP), with linear IgG and C3 at the basement membrane. Both empagliflozin and paliperidone were discontinued. However, the blisters persisted. Treatment included: topical Dermovate and Eumovate ointment for the body and face respectively, alongside oral doxycycline 200 mg and prednisolone 40 mg for a week (reducing by 5 mg/week over 8 weeks). Nevertheless, new blisters continued developing, hence dapsone 50 mg was introduced, with significant improvement. Increasingly, several neurological and psychiatric disorders have been linked with BP, complicating aetiology and management. The underlying mechanism for these associations is not fully understood. Bullous pemphigoid autoantigens BP180 and BP230 are expressed in the central nervous system and it is thought that neurodegeneration may expose antigens to the immune system, generating a cross‐reactive immune response. However, there also appears to be bidirectional causality between BP and neuropsychological conditions. Furthermore, as there was an association of empagliflozin initiation and BP onset, this further complicates the aetiology and presents a potential novel drug cause of BP. This case emphasises the neuropsychological issues associated with managing complex BP cases, a possible novel cause of drug‐induced BP and highlights the likelihood of these issues becoming increasingly prevalent for the future.

## Graphical abstract

This case emphasises the neuropsychological issues associated with managing complex bullous pemphigoid (BP) cases, a possible novel cause of drug‐induced BP and highlights the likelihood of these issues becoming increasingly prevalent in the future.

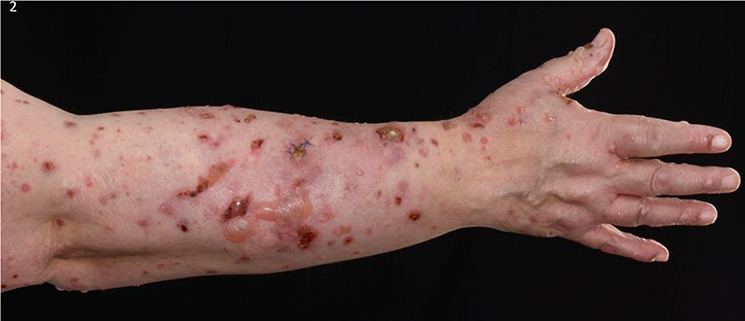

1

**What is already known about this topic?**

- BP shows links with neuropsychological conditions, with multiple sclerosis (MS) indicating the strongest association and schizophrenia patients demonstrating 2.7‐fold‐higher risk against the general population.
- Antipsychotic medications also showed a 1.63‐fold‐increased risk of Bullous pemphigoid (BP).

**What does this study add?**

- The association between BP and SGLT‐2 inhibitors is not fully established. To our knowledge this may be the first documented case of BP associated with empagliflozin, on a background of schizoaffective disorder.
- Only one other case from Japan recorded BP after ipragliflozin, another SGLT‐2 inhibitor.

## 2 INTRODUCTION

Bullous pemphigoid (BP) is an autoimmune blistering skin disorder, commonly affecting older patients with multiple co‐morbidities. There appears to be bidirectional associations between neuropsychological co‐morbidities and BP.^1^ Additionally, these associations also seem to be with the medication used to treat neuropsychological conditions.^1^ One study demonstrated schizophrenia increased the risk of developing BP by 2.7‐fold.^1^ However, further research is required to fully explore the disease mechanism. One theory is that inflammation of the nervous system exposes the BP antigens BP180 and BP230, resulting in a cross‐reactive immune response between neural and cutaneous antigens.^2^ BP patients with neuropsychiatric co‐morbidities have worse outcomes, compared to general BP patients, with increased mortality and poorer compliance to long‐term corticosteroid treatment.^3^ Furthermore, high‐dose corticosteroids are frequently used to treat BP, but are renowned for inducing mood changes.^4^ Clinicians should carefully consider patient co‐morbidities and medication as this could influence the aetiology, presentation and management of BP.^5^ This is likely to be of enduring relevance, given the increase in polypharmacy, co‐morbidities, neuropsychiatric diseases and rising elderly populations, requiring greater holistic care for individuals in the future.^2^

## 3 CASE REPORT

A 59‐year‐old woman, with known schizoaffective disorder, presented with an itchy, blistering rash lasting for 4‐weeks. This started over the neck, spreading to the torso, hands and soles, with mucosal involvement. She had recently been started on empagliflozin, a SGLT‐2 inhibitor for type‐ 2‐diabetes mellitus, 1 month prior to the onset of her cutaneous symptoms. Her past medical history included, agoraphobia, anxiety, hypothyroidism, asthma and hypertension. Her medications comprised of monthly paliperidone injections, adcalD3, alendronate, chlorpheniramine, citalopram, lansoprazole, lisinopril, levothyroxine and lercanidipine.

**FIGURE 1:**
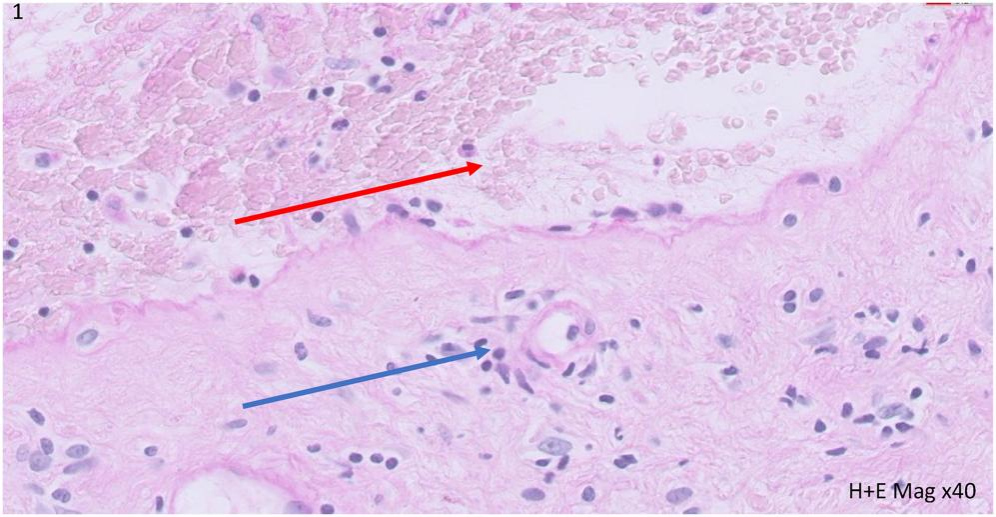
Incisional biopsy from the right forearm of lesional skin (H&E x40mag), depicting sub‐epidermal blister (red arrow) with dermal neutrophils, scattered eosinophils and perivascular inflammation (blue arrow) in keeping with BP

On examination she presented with widespread erosions affecting the whole body and mouth with tense blisters over her hands and soles. Nasoendoscopy demonstrated laryngeal blisters. Serology at presentation was positive for anti‐pemphigoid antibodies, interestingly she had previously tested negative in 2018. Histology demonstrated subepidermal blistering, with a perivascular inflammatory infiltrate and eosinophils (Figure 1). Direct immunofluorescence was characteristic with linear IgG and C3 at the basement membrane.

**FIGURE 2:**
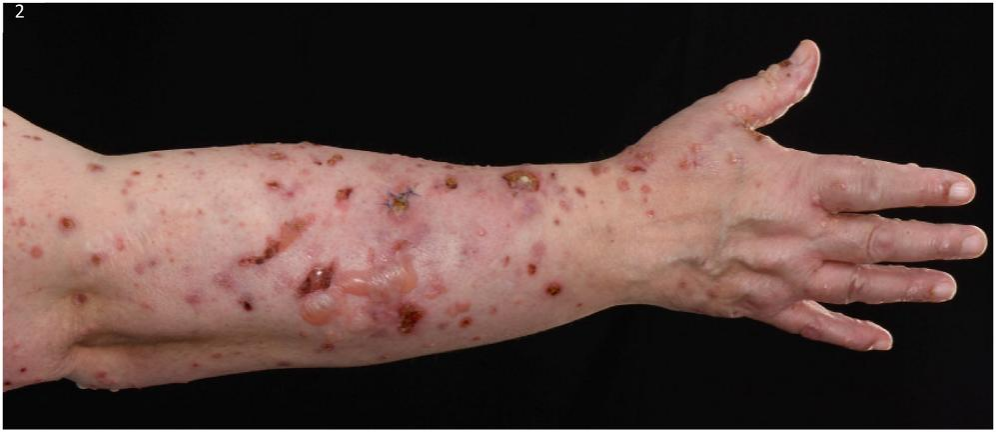
Initial presentation of the extensor surface of the right forearm depicting intact blisters on an erythematous base

**FIGURE 3:**
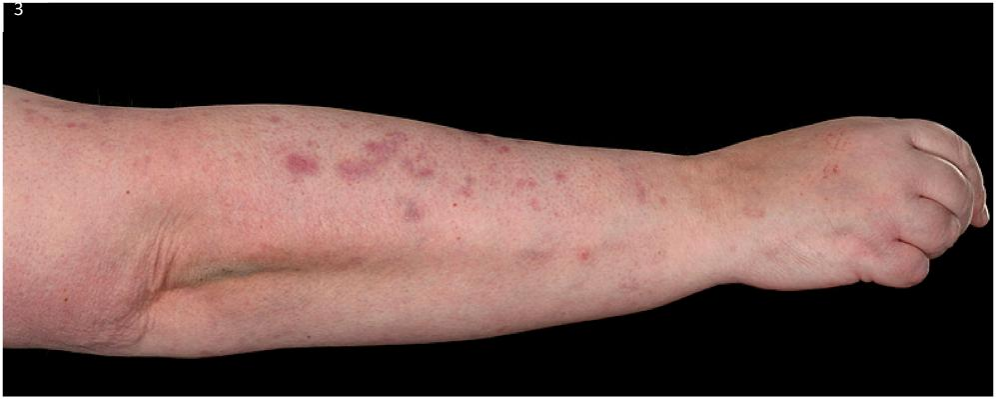
The right forearm post treatment with topical dermovate, oral prednisolone, doxycycline and dapsone 50 mg

**FIGURE 4:**
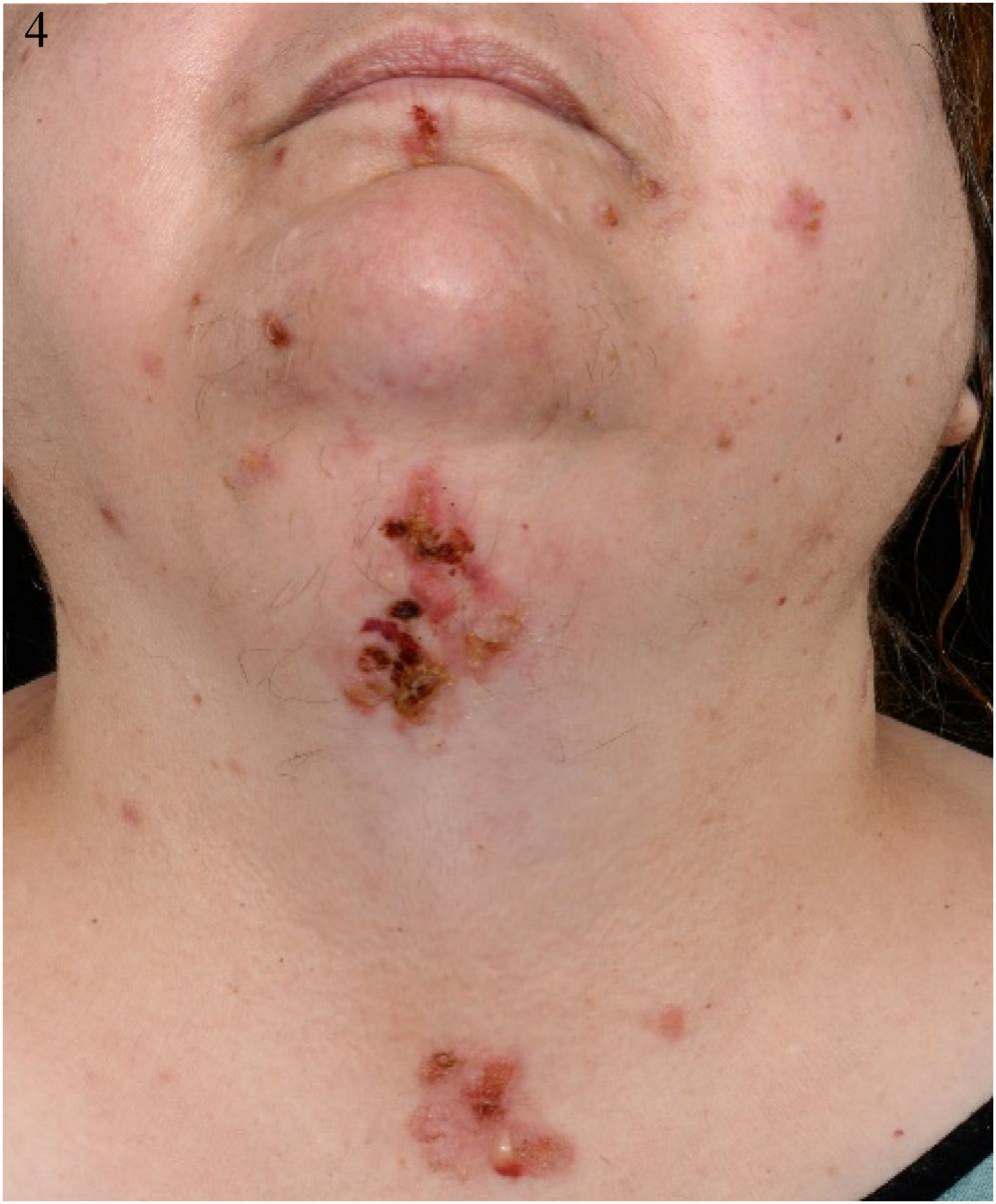
Clinical photographs of the head and neck at initial presentation

**FIGURE 5:**
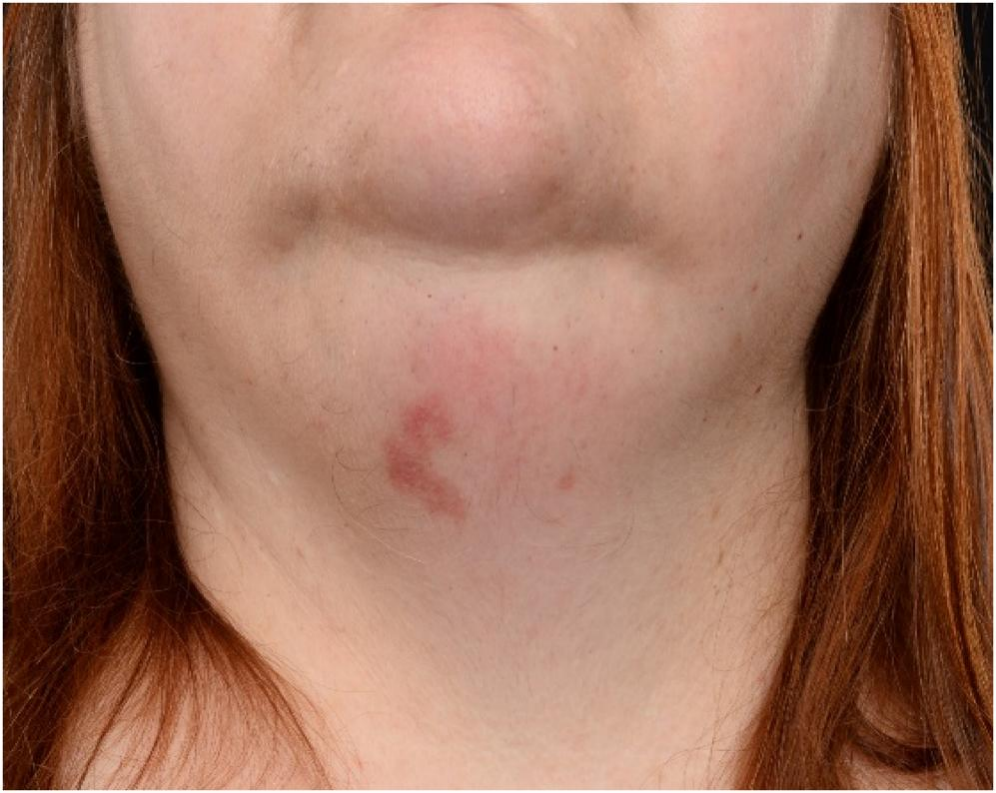
Clinical photographs of the head and neck post‐treatment

Empagliflozin was stopped, as a potential trigger and she was switched to subcutaneous insulin as per the diabetic specialists. She was treated with Dermovate ointment topically once daily under paste bandaging to the trunk and limbs and with Eumovate ointment topically once daily to the face. She was started on doxycycline 200 mg daily orally and prednisolone 40 mg daily orally for a week, reducing by 5 mg/week over 8 weeks. However, new blisters continued developing, hence dapsone 50 mg daily orally was added, resulting in significant improvement (Figures 2, 3, 4, 5).

Post‐discharge she failed to respond to virtual or face‐to‐face appointments, which may be partly attributable to her mental health history. One year later, she was readmitted under general medicine with shortness of breath and concerns of methaemoglobinaemia. The admitting physicians held her dapsone, resulting in recurrence of blisters over her arms. Dermatology advised methaemoglobinaemia was an unlikely cause of shortness of breath as her methaemoglobin level was stable (1.3%), making infection more likely. She responded well to antibiotics with improving saturations and dapsone 50 mg daily was restarted, resolving her blisters. She was discharged however, 1 week later was readmitted with covid pneumonitis, which she sadly died from.

## 4 DISCUSSION

The estimated incidence of BP is 2.5–42.8cases/million/year in Europe^6^ and 4.3/100,000 per person‐years in the UK,^7^ with 1‐year mortality at 26.7%.^6^ Rates appear to be increasing over time, especially in the >60s; this is a trend that is likely to continue with an increasingly ageing population. Additionally, there are recognised associations between neuropsychological diseases and BP, as in our case, so a comprehensive history is paramount.^2^ A Finnish population study demonstrated that neurological co‐morbidities including dementia, Parkinson's, cerebrovascular disorders, epilepsy and multiple sclerosis (MS) all significantly increase the risk of developing BP.^1^ A co‐morbidity of MS confers the highest risk (OR 5.9)^1^ with these patients developing BP 12 years earlier on average, than BP patients without MS.^1^ This association appears to be bidirectional, with a meta‐analysis study showing the risk of developing MS in BP patients as 12.4 times higher than controls.^8^ Furthermore, patients with blistering conditions also showed a higher risk of developing psychiatric disorders (HR 1.30), after correcting for BP medications and diuretics.^6^ Additionally, several psychiatric diseases have also been associated with BP, however, with generally lower risk ratios compared with neurological co‐morbidities. The highest demonstrated associated risks were schizophrenia (OR 2.7), schizotypal and delusional disorders (OR 2.1) and personality disorders (OR 2.2).^1^ The above study also suggested psychiatric diagnoses could precede BP by 7–11 years.^1^ This was further echoed in a Danish study from 1994 to 2016, illustrating patients with pre‐existing psychiatric disorders had a 79% increased risk of developing BP.^6^ This study supported that the highest risk was associated with intellectual disorders (4.18‐fold), schizophrenia and personality disorders (2.01‐fold), independent of psychiatric medications.^6^ A recent large population study showed that BP patients with depression had an 19% increased all‐cause mortality rate compared to those without.^3^ Furthermore, BP patients with a history of anxiety and depression had poorer compliance with long‐term corticosteroid treatment.^3^

The mechanism underlying the association between BP and neuropsychiatric disease is not fully understood. Skin and nervous tissue are derived from the same ectoderm and subtypes of the autoantigen BP230 have been identified in both the central and peripheral nervous system.^6^ The consensus in the literature is that neuroinflammation and neurodegeneration could precipitate a cross‐reactive immune response between neural and cutaneous antigens.^2^

Further research is needed, but evidence suggests that neuropsychiatric disease may influence the immunological phenotype of BP.^9^ A retrospective study found that BP patients with co‐morbid neuropsychiatric disease had a higher seropositivity rate of anti‐BP230 compared to those without (67.7% vs. 36.5%).^9^ Furthermore, these patients had higher circulating eosinophil levels compared to general BP patients, which is suggested to exert neurotoxic effects.^9^ Another study found that BP180 autoantibodies are associated with more severe dementia, as the BP180 enzyme‐linked immunosorbent assay demonstrated inverse associations with cognitive function, measured via the mini mental state examination scores.^10^

Anti‐psychotic medications are a confounder for the association between neuropsychiatric disease and BP.^7^, ^11^, ^12^ The use of antipsychotics increases the risk of BP by 1.63‐fold^11^ and this increases to 3.70‐fold with the use of aliphatic phenothiazines.^7^ A large case‐control study identified a significantly increased risk of BP with the use of periciazine, melperone, haloperidol, biperiden and risperidone.^12^

High dose corticosteroids, are the mainstay of treatment in BP, but are renowned for commonly inducing mood changes, hypomania and mania.^4^ Clinicians should note the psychiatric side‐effects from corticosteroids generally occur in the first 1–2 weeks after initiation and are dose related.^4^, ^13^ Tapering or discontinuation can lead to recovery, although psycho‐tropic medications are often still required.^4^ The BLISTER trial demonstrated doxycycline 200 mg daily effectively controls blisters with a safer profile compared to oral prednisolone.^14^ Furthermore, an experimental study on mice showed doxycycline has antipsychotic‐like effects and could be a safe adjuvant treatment option in schizophrenia.^15^ The underlying mechanisms are unclear, but doxycycline appears to work differently to conventional antipsychotics, which target dopamine and 5‐HT receptors.^15^

Patients with BP also show an increased prevalence of diabetes.^7^ Bullous pemphigoid is mostly associated with DPP4‐inhibitors, which increase the risk of BP by two‐fold^16^ and the highest risk is associated with vildagliptin.^16^, ^17^ Our patient was initiated on empagliflozin, an anti‐diabetic medication, which acts as a reversible inhibitor of SGLT‐2 in the renal tubules. The literature describing the association between SGLT‐2 inhibitors and BP is inconclusive. A retrospective Finnish population study of BP patients aged over 40 years, did not show any increased risk associated with SGLT‐2 inhibitors.^17^ A cohort study of diabetic patients taking SGLT‐2 inhibitors showed a lower risk of BP (HR 0.56), compared to diabetic patients without SGLT‐2 usage, after adjusting for confounders.^18^ Interestingly, a pooled analysis of five randomised controlled trials found that combination therapy with empagliflozin and linaglipin had a similar incidence of adverse events compared to monotherapy and no cases of pemphigoid were reported within the 2895 patients analysed.^19^ However, there is one case report from Japan diagnosing BP 5‐months after ipragliflozin.^20^ No known cases of BP caused by empagliflozin have currently been observed in the literature to date. So far, the Medicines & Healthcare products Regulatory Agency have 9 reported cases of bullous disorders with empagliflozin, with blisters observed but no formal diagnosis of BP documented.^21^ We completed a yellow card for this reaction.^22^

## 5 CONCLUSION

In conclusion, we report a patient with BP, neuropsychological conditions and a possible novel drug association. This case highlights the complexities associated with BP and illustrates the need for clinician awareness of these associations and to use an integrated approach with careful consideration of patients' comorbidities, as this could influence the presentation and management of BP. This is of ongoing relevance and likely to be an enduring issue given the increase in polypharmacy, co‐morbidities, psychoneurological diseases and rising elderly population.

## CONFLICT OF INTEREST

Dr George W. M. Millington is the Editor in Chief of Skin Health and Disease. Dr Sarah M. Dyson is an Associate Editor of Skin Health and Disease.

## AUTHOR CONTRIBUTION

**Sarah M. Dyson**: Conceptualization (Equal); Data curation (Equal); Formal analysis (Equal); Investigation (Equal); Resources (Equal); Writing – original draft (Equal); Writing – review & editing (Equal). **Priya U. Patel**: Conceptualization (Equal); Methodology (Equal); Project administration (Equal); Supervision (Equal); Writing – review & editing (Equal). **Laszlo Igali**: Data curation (Equal); Formal analysis (Equal); Investigation (Equal); Methodology (Equal); Writing – review & editing (Equal). **George W. M. Millington**: Data curation (Equal); Formal analysis (Equal); Investigation (Equal); Methodology (Equal); Project administration (Equal); Supervision (Equal); Writing – review & editing (Equal).

## ETHICS STATEMENT

Not applicable.

## Data Availability

Data sharing is not applicable to this article as no new data were created or analysed in this study.

## References

[1] Försti AK , Jokelainen J , Ansakorpi H , Seppanen A , Majamaa K , Timonen M , et al. Psychiatric and neurological disorders are associated with bullous pemphigoid ‐ a nationwide Finnish Care Register study. Sci Rep. 2016;6(1):37125. 10.1038/srep37125 27845416 PMC5109264

[2] Försti AK , Huilaja L , Schmidt E , Tasanen K . Neurological and psychiatric associations in bullous pemphigoid—more than skin deep? Exp Dermatol. 2017;26(12):1228–34. 10.1111/exd.13401 28677172

[3] Kridin K , Hundt JE , Ludwig RJ , Schonmann Y , Cohen AD . Anxiety and depression predispose individuals to an autoimmune bullous diseases‐bullous pemphigoid: a large‐scale population‐based cohort study. Curr Psychol. 2021:1–11. 10.1007/s12144-021-01396-1

[4] Kenna HA , Poon AW , de los Angeles CP , Koran LM . Psychiatric complications of treatment with corticosteroids: review with case report. Psychiatr Clin Neurosci. 2011;65(6):549–60. 10.1111/j.1440-1819.2011.02260.x 22003987

[5] Poot F . An integrated approach is recommended in bullous pemphigoid. J Eur Acad Dermatol Venereol. 2021;35(10):1919–20. 10.1111/jdv.17600 34533254

[6] Rania M , Petersen LV , Benros ME , Liu Z , Diaz L , Bulik CM . Psychiatric comorbidity in individuals with bullous pemphigoid and all bullous disorders in the Danish national registers. BMC Psychiatr. 2020;20(1):411. 10.1186/s12888-020-02810-x PMC7439544 32819315

[7] Bastuji‐Garin S , Joly P , Lemordant P , Sparsa A , Bedane C , Delaporte E , et al. Risk factors for bullous pemphigoid in the elderly: a prospective case‐control study. J Invest Dermatol. 2011;131(3):637–43. 10.1038/jid.2010.301 20944650

[8] Lai YC , Yew YW , Lambert WC . Bullous pemphigoid and its association with neurological diseases: a systematic review and meta‐analysis. J Eur Acad Dermatol Venereol. 2016;30(12):2007–15. 10.1111/jdv.13660 27599898

[9] Ständer S , Hammers CM , Vorobyev A , Schmidt E , Hundt J , Sadik C , et al. Coexistence of bullous pemphigoid with neuropsychiatric comorbidities is associated with anti‐BP230 seropositivity. J Eur Acad Dermatol Venereol. 2021;35(10):2067–73. 10.1111/jdv.17304 33896070

[10] Kokkonen N , Herukka SK , Huilaja L , Kokki M , Koivisto AM , Hartikainen P , et al. Increased levels of the bullous pemphigoid BP180 autoantibody are associated with more severe dementia in Alzheimer’s disease. J Invest Dermatol. 2017;137(1):71–6. 10.1016/j.jid.2016.09.010 27650606

[11] Liu SD , Chen WT , Chi CC . Association between medication use and bullous pemphigoid: a systematic review and meta‐analysis. J Am Med Assoc Dermatol. 2020;156(8):891–900. 10.1001/jamadermatol.2020.1587 PMC7301306 32584924

[12] Varpuluoma O , Jokelainen J , Försti AK , Turpeinen M , Timonen M , Huilaja L , et al. Drugs used for neurologic and psychiatric conditions increase the risk for bullous pemphigoid: a case‐control study. J Am Acad Dermatol. 2019;81(1):250–3. 10.1016/j.jaad.2019.02.017 30771421

[13] Kluger N , Pankakoski A , Panelius J . Depression and anxiety in patients with bullous pemphigoid: impact and management challenges. Clin Cosmet Investig Dermatol. 2020;13:73–6. 10.2147/ccid.s212984 PMC6982456 32021371

[14] Williams HC , Wojnarowska F , Kirtschig G , Mason J , Godec TR , Schmidt E , et al. Doxycycline versus prednisolone as an initial treatment strategy for bullous pemphigoid: a pragmatic, non‐inferiority, randomised controlled trial. Lancet. 2017;389(10079):1630–8. 28279484 10.1016/S0140-6736(17)30560-3 PMC5400809

[15] Issy AC , Pedrazzi JFC , van Oosten ABS , Checheto T , Silva RR , Noel F , et al. Effects of doxycycline in Swiss mice predictive models of schizophrenia. Neurotox Res. 2020;38(4):1049–60. 10.1007/12640-020-00268-z 32929685

[16] Kridin K , Cohen AD . Dipeptidyl‐peptidase IV inhibitor‐associated bullous pemphigoid: a systematic review and meta‐analysis. J Am Acad Dermatol. 2021;85(2):501–3. 10.1016/j.jaad.2018.09.048 30296540

[17] Varpuluoma O , Jokelainen J , Huilaja L , Tasanen K . GLP‐1 analogs and SGLT2 inhibitors do not increase risk of bullous pemphigoid. J Invest Dermatol. 2021;141(12):2969–72. e1. 10.1016/j.jid.2021.05.015 Epub 2021 Jun 8. PMID: 34116060. 34116060

[18] Ma S , Wu C , Lyu Y , Chou Y , Chang Y . Association between sodium‐glucose co‐transporter 2 inhibitors and risk of bullous pemphigoid in patients with type 2 diabetes: a nationwide population‐based cohort study. J Eur Acad Dermatol Venereol. 2022;36(8):1162–63. 10.1111/jdv.18106 35344615

[19] Watada H , Yamauchi T , Yamamoto F , Taniguchi A , Yarush L , Heilmann C , et al. Safety and tolerability of empagliflozin and linagliptin combination therapy in patients with type 2 diabetes mellitus: a pooled analysis of data from five randomized, controlled clinical trials. Expet Opin Drug Saf. 2020;19(9):1193–202. 10.1080/14740338.2020.1782884 32552153

[20] Ikehara K , Uchino H , Kakumae Y , Miyashita's N , Yoshino H , Miyagi M , et al. A case of bullous pemphigoid associated with the administration of ipragliflozin, a sodium‐glucose cotransporter 2 inhibitor. J Jpn Diabetes Soc. 2018;61(2):59–63.

[21] Medicines and Healthcare products Regulatory Agency . https://info.mhra.gov.uk/drug‐analysis‐profiles/dap.html?drug=./UK_EXTERNAL/NONCOMBINED/UK_NON_000278755963.zip%26agency=MHRA. Accessed 6 June 2022.

[22] Medicines and Healthcare products Regulatory Agency . Yellow card scheme. https://yellowcard.mhra.gov.uk. Accessed 6 June 2022.

